# African American Emerging Adult Perspectives on Unintended Pregnancy and Meeting Their Needs With Mobile Technology: Mixed Methods Qualitative Study

**DOI:** 10.2196/21454

**Published:** 2020-10-21

**Authors:** Lucy Annang Ingram, Crystal Stafford, Quentin McCollum, McKenzie Isreal

**Affiliations:** 1 Department of Health Promotion, Education, and Behavior Arnold School of Public Health University of South Carolina Columbia, SC United States; 2 Department of Veteran Affairs Durham VA Medical Center Durham, NC United States; 3 College of Social Work University of South Carolina Columbia, SC United States

**Keywords:** unintended pregnancy, emerging adult, African American, mobile technology, pregnancy, teenage years, health promotion, mobile, sexual health

## Abstract

**Background:**

In the United States, a disproportionate number of unintended pregnancies occur among African Americans, particularly those in their later teenage years and early 20s. Mobile technology is becoming more ubiquitous as a method for health promotion; however, relatively little research has been done with this population to determine their perspectives about unintended pregnancy, the potential of successfully using mobile technology to prevent unintended pregnancy, and the content of such programs.

**Objective:**

The purpose of this study was to obtain the perspectives of African American emerging adults about unintended pregnancy and the use of mobile technology to reduce unintended pregnancy rates.

**Methods:**

Focus groups and interviews were conducted with 83 African Americans, aged 18-21 years. Data were analyzed using an open coding process. Emergent codes were then added as needed, and themes and subthemes were identified.

**Results:**

Participants cited the social environment and lack of education as primary reasons for disproportionate rates of unintended pregnancy. They noted that unintended pregnancy is an important issue and that they desire more sexual health information. They enthusiastically supported mobile technology as a means to communicate unintended pregnancy prevention programming and offered many suggestions for program content, look, and feel.

**Conclusions:**

Young and emerging adult African Americans want and need sexual health resources, and a mobile-based platform could be widely accepted and address needs to lower disproportionate rates of unintended pregnancy. An essential next step is to use these findings to inform the development of a mobile-based unintended pregnancy prevention and sexual health program prototype to determine feasibility.

## Introduction

In the United States, unintended pregnancy is common. In 2011, the rate of unintended pregnancies was 45% [1,2]. Most notably, unintended pregnancy rates are highest among older teens and women in their early 20s [3,4]. Among these, a disproportionate number occurs among racial and ethnic minorities. Of all unintended pregnancies in the United States in 2011, 38% were among Whites, 50% were among Hispanics, and 64% were among African Americans [1]. Although unintended pregnancy rates have been on a steady decline nationally, the disparity among racial and ethnic minorities warrants further attention.

Unintended pregnancy, particularly among youth, can have health consequences for the mother and child, educational consequences such as reduced probability of obtaining a college degree, and economic consequences such as reduced lifetime earnings and taxpayer burden [5]. Other social consequences, such as shame and disconnection from friends and family, are prevalent among this population [6]. The reasons for these disproportionately high rates are varied and include structural inequalities, negative attitudes about contraception, limited partner communication, and inadequate knowledge about the effectiveness of different contraceptive options or about how to use specific methods [2,7-13].

When examining rates by region of the United States, racial disparities in unintended pregnancy rates are even more pronounced in the South. In particular, young adult African American women have some of the highest rates of unintended pregnancy in South Carolina [14]. South Carolina’s birth rate is 21.7 births per 1000 among women aged 15-19 years [14]. The 2013 birthrate for 18–19-year-old African American women in South Carolina was 70.4 per 1000, whereas the rate among their white counterparts was 52.3 per 1000 [15]. These data reveal the significant need to address unintended pregnancy in South Carolina and among African American young adults in particular.

Young African Americans are just as likely to use the internet as young whites, and nearly all African Americans between the ages of 18 and 29 years have been found to use at least one social media app [16]. With the majority of the population having access to a smartphone, the use of mobile technology can play a significant role in promoting sexual health and ultimately reducing unintended pregnancies. In 2016, Mangone et al [17] published their research with analysis of over 6800 mobile apps related to pregnancy prevention. Overall, the investigators found that, while there were some innovative, creative, and educational apps in the market, very few used evidence-based practices, had information about modern contraception methods, and were tailored to a particular race or ethnicity. Moving forward, if mobile technology is to be a method of addressing unintended pregnancy, the products should be enhanced so that the most accurate and effective information is made available to the audiences that could greatly benefit from access to unintended pregnancy prevention and sexual health promotion information. To address this gap in the literature, we conducted a qualitative study of African Americans, aged 18-21 years, to obtain their perspectives about using mobile technology to prevent unintended pregnancy among young adults.

## Methods

### Study Participants

This study used focus group and interview methodology to determine perspectives on using mobile technology to reduce unintended pregnancy among young adult African Americans. Study participants consisted of African American young adults, aged 18-21 years. Additionally, efforts were made to recruit both parenting (those who were pregnant or had a child) and nonparenting young adults (those who were not pregnant and never had a child). Recruitment flyers were developed that incorporated images representative of the intended audience (eg, African American young adults, pregnant African American women with a male partner, teachers, health care professionals), a description of the study purpose, eligibility criteria, and staff contact information including a land line phone number, text-enabled phone number, and email address. Purposive sampling was employed whereby participants were recruited using a variety of methods, maximizing the locations where potential study participants were likely to be found. For example, flyers were posted in high-traffic areas for African American young adults and circulated among networks such as college professors and college student organizations (at both predominantly white institutions and historically Black colleges and universities), health educators, community groups, churches, public libraries, family planning clinics, social media websites (Facebook and twitter), and word of mouth. Community events where intended audience members congregate, such as sports tournaments and a local step show, were also venues for recruitment. Potential participants were screened for eligibility and asked their availability to attend a 90-minute focus group. We also employed the strategy of tentatively scheduling focus group meetings as we found that several potential participants were more amenable to having a scheduled “appointment” rather than having to be contacted later to determine their ability to attend a group on some future date. Additionally, spontaneous interviews were conducted in the event that the recruiter and eligible participant had availability at the time of the initial meeting. Eligibility criteria for the participants were: African American race, age 18-21 years, ability to attend a 90-minute focus group meeting, and English as the primary language. Potential participants were sent study reminders (via phone conversation, voice mail, email, or text message) at least 1 week, 3 days, and the day prior to, as well as the day of, the scheduled focus groups to help facilitate their attendance. Participants were enrolled January 2016 through June 2016. All study procedures were reviewed and approved by the University of South Carolina institutional review board.

### Instrumentation

A brief, self-administered, demographic and sexual health questionnaire was developed by the lead researcher to obtain a profile of the study participants. Demographic measures included age, sex, race, ethnicity, marital status, educational level, employment status, and sexual orientation. Sexual health information included a range of questions to help describe participants’ sexual background as well as sexual health history. For example, questions were included about age of sexual debut, whether or not participants had children, as well as when or if they ever received formal sexual health instruction, the content of that instruction, and their perception of where most African American youth receive sexual health and teen pregnancy prevention information.

A focus group guide was developed to standardize the conduct of focus group sessions. Open-ended questions were developed by the first author based on the research aims of the study. The questions were guided by a review of extant literature in unintended pregnancy prevention, sexual health promotion, and mobile technology. Based on this, the focus group questions were organized into 4 categories: importance of teen pregnancy prevention, technology as a method to promote teen pregnancy prevention, content of a technology-based program to promote teen pregnancy prevention, and design and format of a technology-based program to promote teen pregnancy prevention. The lead author is a public health researcher and professor who has had over 20 years’ experience in the development, implementation, and analysis of focus groups and qualitative instruments. Focus group questions were pretested with members of the intended audience to ensure content validity. Based on the feedback received during the pretest, the focus group questions were refined and finalized.

### Procedures

Participants were invited to attend a focus group to share their thoughts about how young adult African Americans use mobile technology for sexual health information. The researcher and a research assistant obtained training about policies for the protection of human subjects in research, and at least one of the staff members was available at each focus group meeting to obtain informed consent. Additionally, the researcher trained the research assistant in best practices for conducting and moderating successful focus groups including information about note taking, encouraging participation, and navigating roadblocks. Participants were asked to review a copy of the informed consent document as a staff member reviewed it with them. Participants were encouraged to ask questions during and after the document review, were then asked to sign the consent form indicating their approval, and were given a copy of the consent document for their records. Once participants were enrolled into the study, they were asked to complete an anonymous self-administered paper-and-pencil survey. In order to limit feelings of anxiety regarding responses to sensitive questions, after the staff member provided participants their surveys, participants completed them, were asked not to attach their name to the survey, and were then asked to place the completed survey in a sealed envelope that would not be opened until the end of the recruitment day. All participants received a US $15 cash incentive for their participation. Focus groups were scheduled for days and times that were convenient to study participants and ranged from mid-day to late evening, any day of the week. In the event that the staff member and eligible participant had availability at the time of the initial meeting, spontaneous interviews were conducted. Meetings or interviews were conducted by a member of the research team who had been trained to moderate focus group discussions and conduct interviews.

### Data Analysis

Focus group meetings were digitally audio-recorded. Audio recordings were reviewed and analyzed using the memoing technique [18], which focused on identifying repeated responses that emerged from participants as well as those that emerged and represented new ideas and notions. Memos allowed for coding and categorizing data as well as allowing the researchers to scrutinize the data to explore hypotheses, relationships between concepts, and explanations that emerged from the data. A draft of the codebook to categorize participant responses was established using an open coding process [19] using the discussion guide as a framework. Emergent codes were then added as needed. The data were triangulated using transcripts, field notes, and memos and analyzed by two independent researchers using inductive, semantic thematic analysis. Any discrepancies were resolved by consensus. Survey data were analyzed using SPSS version 23, and descriptive statistics were performed.

## Results

### Demographic Characteristics and Sexual Health Information

A total of 83 young adults participated in the study, and 13 focus groups and 7 interviews were conducted. Participants ranged in age from 18 to 21 years (mean age 19.5 years) and were primarily African American (82/83, 99%) and of non-Hispanic ethnicity (79/83, 95%). One participant identified as multiracial. Of the participants, 98% (81/83) reported never being married, and 52% (43/83) had some high school education or had earned at least a high school diploma. Of the participants, 93% (77/83) identified as heterosexual, with 7% (6/83) identifying as gay, lesbian, or bisexual.

Regarding sexual behaviors, 83% (69/83) of participants had ever had consensual sex, with 16 years as the average age of first sexual experience. Among those who were sexually experienced, 15% (11/83) had ever been or ever gotten someone pregnant. As it relates to formal sex education, the majority of participants (80/83, 96%) had received some form of sex education before age 18, with 75% (62/83) having their first instruction before entering high school. As part of their sex education, participants identified having learned about abstinence until marriage, birth control, condoms, and sexually transmitted infections (STIs), with the overwhelming majority having received information about condoms (76/83, 95%) and STIs (76/83, 95%). When asked about where they think most African American youth receive information about teen pregnancy prevention, over half of the sample identified family members (47/83, 57%) and school (45/83, 54%) as the most popular sources. The next most popular sources of information were friends and social media at 45% (37/83) and 41% (34/83) of participants, respectively. The sources identified least often were music (8/83, 10%), flyers/pamphlets (7/83, 8%), movies (5/83, 6%), newspapers/magazines (3/83, 4%), and church (1/83, 1%). When asked about whether they think that African American youth receive enough accurate information about teen pregnancy prevention, most participants (60/83, 73%) responded “no” or “not sure” (see Table 1).

**Table 1:** Demographic and sexual health characteristics (N=83).

Characteristics		Results
Age (years), mean (range)		19.5 (18-21)
**Sex, n (%)**		
	Male	34 (41)
	Female	49 (59)
**Race, n (%)**		
	African American	82 (99)
	Multiracial	1 (1)
**Ethnicity, n (%)**		
	Non-Hispanic	79 (99)
	Hispanic	1 (1)
**Marital status, n (%)**		
	Single/never married	81 (98)
	Married	0 (0)
	Divorced	0 (0)
	Other	2 (2)
**Highest level of education, n (%)**		
	Some high school	8 (10)
	High school diploma	35 (42)
	Some college	37 (45)
	Associate’s degree	1 (1)
	Bachelor’s degree	2 (2)
	Some graduate school	0 (0)
	Graduate degree	0 (0)
**Sexual orientation, n (%)**		
	Heterosexual	77 (93)
	Gay or lesbian	5 (6)
	Bisexual	1 (1)
**Sexual initiation, n (%)**		
	Yes	69 (83)
	No	14 (17)
Age of sexual initiation (years), mean (range)		16 (12-18)
**Ever been pregnant/gotten someone pregnant, n (%)**		
	Yes	11 (15)
	No	60 (83)
	Not sure	1 (7)
**Formal sex education, n (%)**		
	Yes	80 (96)
	No	3 (4)
**Grade of earliest sex education, n (%)**		
	Elementary school (1st-5th grades)	19 (23)
	Middle school (6th-8th grades)	43 (52)
	High school (9th-12th grades)	17 (21)
	College	1 (1)
**Content of sex education, n (%)**		
	Abstinence until marriage	58 (73)
	Birth control	61 (77)
	Condoms	76 (95)
	Sexually transmitted infections	76 (95)
**Common sources of African American youth teen pregnancy prevention information, n (%)**		
	Family member	47 (57)
	School	45 (54)
	Friends	37 (45)
	Social media	34 (41)
	Health care provider	33 (40)
	Television	20 (24)
	Websites	13 (19)
	Music	8 (10)
	Flyer/pamphlet	7 (8)
	Movies	5 (6)
	Newspaper/magazine	3 (4)
	Other (church)	1 (1)
**African American youth receive enough accurate teen pregnancy prevention information, n (%)**		
	Yes	22 (27)
	No	45 (55)
	Not sure	15 (18)

### Perspectives on Teen Pregnancy and Prevention

#### Disproportionately High Rates of Pregnancy Among African American Older Adolescents are Due to Social Environment and Lack of Education

Participants did not seem to be surprised that, while rates of unintended pregnancy among youth and young adults have declined as a whole, we continue to see rates high among African Americans, particularly those aged 18-19 years. A variety of reasons for this statistic were proffered, with many of them centered around 2 notions: the social environment and lack of education.

As one participant stated, “I would think their social environment [is the reason why the rates are high among African Americans]. It depends who they are influenced by either at home or out in the community or even just what they see out in the community. They just think that it’s fine and it’s cool. They never talk about the right way to have sex…they only see about the wrong ways.” Another participant explicitly identified messages in music as a culprit. He stated that the messages in music promote “that type of behavior,” presumably unprotected sex. Other participants described the environs that are changing for that age group in that many may be going from high school to the “freedom” of college and the feeling that they are adults and can make decisions such as having sex without concern for parents’ judgment or oversight. As one participant noted, “18-19 is that transitional period, and when you come to college, you can do whatever you want to do.”

In addition to one’s environment as a causal factor, several participants suggested that the reason for the disproportionate rates was lack of education. As one participant stated, “Nobody is teaching about it, and there is little guidance. What little you do learn from school, you don’t always take it to heart, and teachers don’t answer all the questions, and you’re not comfortable talking to parents, so…” As stated by another participant, “There is definitely a lack of education about sex and preventing pregnancy,” so she was not surprised by the statistic.

#### Teen Pregnancy is an Important Issue

Overwhelmingly, participants noted that teen pregnancy is an important issue. Discussions about why it is important centered around the idea that young people should try to preserve their youth and with a child comes responsibilities that are vastly different and adult-like. According to one parenting participant, “Because now that I have a child, it is not easy, I wouldn't want anyone else who is not ready or prepared…I know I wasn’t…I had to rush and get prepared…and like in high school, you got school work, and if you have a job and all that…it’s a lot to deal with.” Another participant stated, “They haven't had time to develop yet, and they're still learning about themselves.” Other comments included, “you haven’t started your life so how can you start another life?” “you’re still a child,” teens should “wait until they are situated,” and “babies shouldn’t raise babies.”

#### What I Didn’t Learn About Teen Pregnancy Prevention

There was a variety of responses to the question about what participants did not learn about pregnancy prevention when they were in school, and in fact, responses more broadly reflected what they did not learn about sexual health in general. As it relates to pregnancy prevention, one parenting female participant noted that she did not learn about how hard it would be, and as she stated, “what all I had to go through.” Others noted that they did not learn about how to put on a condom, abstinence, birth control, and the withdrawal method. More contextual responses were about practical things and less about clinical outcomes or biological processes. For example, one participant stated, “I wish that when I learned about pregnancy prevention and sex ed and stuff, they woulda told me that masturbation is okay. I mostly do it now to keep myself out the bull*$#!” indicating the need to learn about alternatives to seek pleasure. Another participant stated, “What they didn’t say was while you’re learning, your mindset will be totally different [than] when you’re in that position, like when you’re in that moment, your mind is not there”. Responses that are applicable to what was not learned about sexual health in general included the consequences of having multiple sex partners, the high level of peer pressure in high school to engage in sexual activities, STIs, and sexual orientation (other than heterosexuality).

#### Preferred Format of a Pregnancy Prevention Program

Participants identified several formats for a pregnancy prevention program that reflected their preferred type of interaction and platform of interaction. Some participants preferred individual interaction while others preferred a group setting for interaction. Those preferring an individual format described the benefits of confidentiality and privacy. For example, one participant stated, “I would like an individual format so you can be more comfortable and have no judgment from others.” For those who preferred a group format, reasons for this included modeling behavior and ability to glean from others’ experience. One participant noted, “In a group, if your friends think that preventing pregnancy is cool, then you’re gonna think it is too.” Another participant stated, “If you have the group discussion, then it’s like, if you have the right people in there, it’s like the influence of it is just going to change their mind…” Yet another participant commented that they would prefer a group so that you “could hear other peoples’ stories.” In instances where the initial responses of a group were divergent, at the end of the discussion, each perspective could see the benefits of the other (even if they still preferred their own).

Regarding the use of mobile technology as a format, the majority of participants liked the idea of having a mobile platform to provide pregnancy prevention information. Reasons for this included “everyone is on their cell phone,” “youth spend a lot of time on their social media,” “I’m mostly on my phone…but not on a computer,” and an app “would be good so you can express yourself privately…and I’m on my phone all the time.” When further prompted about specific aspects of a mobile program, participants noted that it should be brief, interactive, have little text, be entertaining, and allow for access across multiple sittings (rather than needing to be completed at one point in time).

### A Mobile Technology Program for Pregnancy Prevention

#### Program Content — Topics

When asked about the topics that should be included in a mobile technology program for pregnancy prevention among African American youth, participants offered a wide variety of responses. Similar to responses about what they didn’t learn about pregnancy prevention, participants tended to respond about interest in information about sexual health content in general. Frequently suggested topics included condom use and birth control. Additional interests included information about relationship dynamics whereby participants mentioned consent, modeling ways to say “no,” how to communicate with partners about birth control, and how to handle abuse. Several participants indicated wanting to know about sexual orientation and LGBTQ issues with several groups openly debating about whether pregnancy prevention would be applicable to sexual minorities. When prompted, participants also noted that substance use would be a topic of interest, particularly as it relates to its influence on decision making.

#### Program Content — Messages

Study participants had very insightful ideas about program messages. Some suggested that engaging in safer sex, having planned birth control, and waiting (until a certain age, until you’re responsible, until you have a connection, or until marriage) should be clear messages. Others took a more inspired route providing suggestions such as “It goes down in the D.M.” (a reference to the social media site Instagram’s direct message feature…as well as a popular rap lyric), “Open books, not legs,” “Be yourself,” “A real man knows that she’s worth the wait,” and “Use the strap if you don’t want the clap.” Conversely, participants also offered suggestions for what not to include such as abstinence messages, information about the pull-out method, and the message, “don’t have sex.”

#### Gender Dynamics

When asked about whether men and women should receive the same program content, responses were mixed. Some participants stated that the same information should be given to both men and women. One participant’s rationale was that, in fact, men should have information that is pertinent to women so that men can know how to “help her out or make her feel better.” According to another participant, “the consequences are the same for both genders so, yeah, they should get the same information.” Participants who suggested that men and women receive different information highlighted interesting perspectives on gender dynamics and gender roles. For instance, one male participant stated, men should know “never have unprotected sex even if she says she’s on the pill,” and another male participant stated that, “men should know how to think with the head on their shoulders.” According to one female participant, men should know “your body count doesn’t make you a man.” Another suggestion from a female participant was, “if you’re serious about a partner, get tested together.”

#### Parenting Dynamics

Participants provided different messages about whether parenting and nonparenting youth should receive the same program content. For those who thought parenting and nonparenting persons should receive the same information, many reflected on the fact that even though someone may have had a child already, they still may want to and need to know information about how to prevent a subsequent unintended pregnancy. As one participant stated, “I feel like, well the people who do already have kids and everything, it be some stuff they don't know that they didn't get or receive so they should all get the same information.” For those whose perspective was that different information should be provided, they pointed out that parenting teens could serve as models and tell others “what it’s about and how it is.”

#### Program Design

Participants provided their ideas about the optimal design for a mobile technology program for pregnancy prevention for African American older adolescents. Participants provided feedback about colors (“it should bold,” “there should be bright colors”), images (“there should be real pictures, but they shouldn’t be too graphic” and “the people should be attractive”), music (if any music, soft music is preferred so as not to be distracting), and videos (preferably tutorials such as how to put on a condom). Some suggested alternatively making the look of the program an interactive feature whereby users could tailor it to their own style.

Participants also described different features that could enhance participation or interest. Suggestions included offering some type of incentive to download the program, connecting it to social media (to not only garner more followers and participants but also connect to modes of communication that they already use), integrating endorsements (these could be from celebrities, adult mentors, or even friends [or relatable others] who have used the program and liked it), and making it fun and attractive.

## Discussion

### Principal Findings

Based on national trends, unintended pregnancy is on the decline [1], which could suggest that the topic is of waning to little interest to young and emerging adults. However, we found the contrary to be true. Nearly 90 young people were recruited to participate in this study to provide their perspectives about unintended pregnancy. Our findings reveal that they are indeed concerned with the trends, particularly those highlighting disparities among African Americans, and have many ideas for how to promote unintended pregnancy prevention and overall sexual health in their communities.

Commonly, respondents found it difficult to divorce unintended pregnancy prevention from overall sexual health. Many suggestions that respondents provided about topics and messages of interest reflected conventional prevention content such as promoting condom and contraception use [20]. Additional suggestions highlighted contextual dynamics of engaging in sexual behavior such as relationship dynamics, emotional challenges involved with young adulthood and sexual relationships, and decision making (eg, whether or not to have sex, whether or not to use contraception). These findings reflect other studies wherein researchers have elicited perspectives of youth, young adult, or influential stakeholders about adolescent pregnancy prevention program content [21,22] that may be precursors to any actual behavior or complementary factors that co-occur with sexual behavior. It was also clear that, when asked about their own sexual health education, participants noted some clear deficiencies. The move to adopting comprehensive sex education programs is controversial in some states and communities [23-25], with South Carolina as no exception [26]. Participants’ lack of exposure to consistent, medically accurate, sexual health information could be reflective of this context. These findings support developing programs that include comprehensive messages to promote sexual health and improve on sexual health knowledge among young and emerging adults.

The idea of using mobile technology to promote unintended pregnancy prevention and sexual health was widely supported. None of the participants suggested that such a platform would not work and in fact enthusiastically provided suggestions ranging from key features, interface look and feel, and compatibility with existing social media platforms. Other researchers have reported about the vast opportunity that mobile apps have for reaching youth and young adults with sexual health education content [17,27-32]; however, there remains a gap in the success of these programs among racial and ethnic minority populations that suffer disproportionately from poor health consequences. More work must be done to determine the best content, prevention strategies, and interactive functionality that these youth will be most receptive to on mobile platforms.

A significant finding that we want to highlight is the fact that study participants were not surprised by the fact that African American young adults outpace all others in unintended pregnancy rates and that this racial health disparity continues to persist. Either because this is a part of their reality or because they are accustomed to experiencing health outcomes different from majority populations, the researchers found this slightly concerning. Some respondents commented that there does not need to be different (read: tailored) programs because “we all experience these public health challenges”; however, a majority of respondents noted that it would be great to have programs and systems that they felt were “for them.” Future research could do well to explore this notion further.

### Limitations and Strengths

Our study is subject to several limitations. One limitation is the sampling technique used for the study. We used purposive sampling to obtain our study sample. In some regards, while this approach was successful in garnering eligible study participants, it could introduce bias and limit generalizability of study findings. Another limitation is the terminology used to discuss the focus of the project. Many of the study documents included the term “teen pregnancy”; however, due to the age range of the study population exceeding the teen years we also used the term “unintended pregnancy.” The vast majority of teen births are among women aged 18-19 years, and rates of unplanned pregnancy are highest among those in their late teens and early 20s [3,4]. While we are confident that the interchangeable use of the terms did not likely negatively impact our study findings, the discrepancy is noteworthy.

Strengths of the study should also be noted. Our ability to recruit a community sample of more than 80 18–21-year-old African American participants to conduct qualitative assessments is significant. This population is frequently characterized as “vulnerable” and “difficult-to-reach,” which often translates to an excuse for a researcher’s inability to obtain their active participation. Our recruitment efforts were successful because they were targeted to the spaces and places where the intended audience was likely to be found. Additionally, about the sample, 40% was comprised of men. Similar to the aforementioned characterization of African Americans, discussions with men about unintended pregnancy are limited. Most often, the topic is left the responsibility of women, with little consideration for the perspectives of male partners. More opportunities to elicit the perspectives of male partners are needed.

### Conclusions

A qualitative approach proved to be useful for assessing young people’s perspectives on unintended pregnancy as well as their thoughts about mobile technology as a viable platform for health promotion. While it was clear that many participants had received some form of sexual health education, many still expressed that young and emerging adults want and need additional sexual health resources and that a platform, such as one that is mobile-based, could be widely accepted, address needs to lower disproportionate rates of unintended pregnancy, and address barriers related to access to health information. An essential next step to build upon this study is to use these findings to inform the development of a mobile-based unintended pregnancy prevention and sexual health program prototype. Based on our findings, we offer the following recommendations to developers, researchers, and program planners working in this area:

1. Integrate sexual health content (highlighting unintended pregnancy) into a mobile app.
2. African American young adults recognize unintended pregnancy as an important issue. Develop content and messages that reflect this.
3. Allow for participants to navigate the app on their own as well as allow group interactive features.
4. Provide real-life scenarios such that it is clear that decision making can be challenging when one is in a compromised or heat-of-the-moment situation.
5. Provide authentic reasons why unintended pregnancy at a young age can be difficult. Highlight stories from teen parents who can share their experiences.
6. Provide information on a variety of topics including condoms, contraception, relationship dynamics, emotional dynamics of young adulthood, sexual decision making, and sexual orientation. Our participants did not support including messages about abstinence or less effective contraceptive methods.
7. Ensure that the design of the interface is modern and reflective of images and language that resonate with the demographic.
8. Ensure compatibility with social media.

Given our success of obtaining young and emerging adult perspectives for the current study, it would be advisable to include young people in the prototype development process as well. Once a prototype is developed, pilot testing for uptake and acceptability as well as outcomes such as improvements in knowledge and behavior would be appropriate before larger scale-up.

## Abbreviations

STI: sexually transmitted infection
